# Risk of selection bias in randomised trials

**DOI:** 10.1186/s13063-015-0920-x

**Published:** 2015-09-10

**Authors:** Brennan C. Kahan, Sunita Rehal, Suzie Cro

**Affiliations:** Pragmatic Clinical Trials Unit, Queen Mary University of London, E1 2AB London, UK; MRC Clinical Trials Unit at UCL, WC2B 6NH London, UK

**Keywords:** Randomised controlled trial, Clinical trial, Selection bias, Randomisation procedure

## Abstract

**Background:**

Selection bias occurs when recruiters selectively enrol patients into the trial based on what the next treatment allocation is likely to be. This can occur even if appropriate allocation concealment is used if recruiters can guess the next treatment assignment with some degree of accuracy. This typically occurs in unblinded trials when restricted randomisation is implemented to force the number of patients in each arm or within each centre to be the same. Several methods to reduce the risk of selection bias have been suggested; however, it is unclear how often these techniques are used in practice.

**Methods:**

We performed a review of published trials which were not blinded to assess whether they utilised methods for reducing the risk of selection bias. We assessed the following techniques: (a) blinding of recruiters; (b) use of simple randomisation; (c) avoidance of stratification by site when restricted randomisation is used; (d) avoidance of permuted blocks if stratification by site is used; and (e) incorporation of prognostic covariates into the randomisation procedure when restricted randomisation is used. We included parallel group, individually randomised phase III trials published in four general medical journals (*BMJ*, *Journal of the American Medical Association*, *The Lancet*, and *New England Journal of Medicine*) in 2010.

**Results:**

We identified 152 eligible trials. Most trials (98 %) provided no information on whether recruiters were blind to previous treatment allocations. Only 3 % of trials used simple randomisation; 63 % used some form of restricted randomisation, and 35 % did not state the method of randomisation. Overall, 44 % of trials were stratified by site of recruitment; 27 % were not, and 29 % did not report this information. Most trials that did stratify by site of recruitment used permuted blocks (58 %), and only 15 % reported using random block sizes. Many trials that used restricted randomisation also included prognostic covariates in the randomisation procedure (56 %).

**Conclusions:**

The risk of selection bias could not be ascertained for most trials due to poor reporting. Many trials which did provide details on the randomisation procedure were at risk of selection bias due to a poorly chosen randomisation methods. Techniques to reduce the risk of selection bias should be more widely implemented.

## Background

Well-conducted randomised controlled trials (RCTs) are viewed as the ‘gold standard’ for comparing different interventions, as they are not subject to the same confounding as non-randomised studies. Randomisation (when performed correctly) guarantees that, on average, treatment groups will be well-balanced for both known and unknown factors, thus ensuring an unbiased estimate of the treatment effect. However, this guarantee of balance only occurs when randomisation is performed correctly; that is, when the probability of a patient being enrolled does not depend on the probability of them being assigned to a particular treatment group. Otherwise, the trial will be at risk of selection bias.

Selection bias occurs when those in charge of the recruitment or enrolment of patients (recruiters) selectively enrol patients into the trial based on what the next treatment allocation is likely to be. For example, if a recruiter believes the next allocation will be the intervention, they may wait to enrol a very sick patient, as they do not want to ‘waste’ an intervention allocation on a relatively healthy patient who is less likely to need it. This can lead to substantially biased estimates of treatment effect and misleading conclusions [1–9]. Selection bias is primarily an issue in RCTs where patients are enrolled sequentially (rather than all at once), and when recruiters can decide whether or not to enrol each eligible patient.

Selection bias depends on the ability of the recruiter to guess with greater than 50 % probability what the next treatment allocation will be. This could happen if, for example, the recruiter had access to the randomisation list, and knew what treatment each patient would be assigned to before enrolling them. For this reason, allocation concealment has been recommended as an essential tool for RCTs [7, 10, 11]. Allocation concealment is ‘*a technique used to prevent selection bias by concealing the allocation sequence from those assigning participants to intervention groups, until the moment of assignment*’ (http://www.consort-statement.org/resources/glossary).

However, even if appropriate allocation concealment is used and recruiters do not have access to allocation list before enrolling patients, they still may be able to guess what the next allocation will be based on the method of randomisation. This is primarily a concern in unblinded trials, where investigators are aware of each patient’s treatment assignment. However, this could also be an issue in blinded trials where there is a risk of unblinding: for example, because side effects may occur for patients in one arm only. Brown et al*.* [12] found that 16 % of the investigators they surveyed had admitted to keeping track of the number of allocations to each treatment group in an attempt to predict the next allocation. This type of behaviour has led to the identification of a number of RCTs which may have been affected by selection bias [13–15]. Because the possibility of selection bias is rarely reported in trial publications, it is likely that the overall problem is underreported. Selection bias can have serious implications for patient healthcare; the distortion of trial results could lead ineffective interventions appearing helpful or harmful interventions appearing safe. The purpose of this article is to highlight some simple methods to prevent selection bias, and assess how often these methods are being used in practice.

## Methods

We begin by describing the problem of selection bias in more detail, and then highlight some simple methods to reduce the risk (summarised in Table 1). We assume that the trial is open-label (unblinded), and that there is adequate allocation concealment.

**Table 1:** Methods to reduce the risk of selection bias in non-double-blind clinical trials*

Techniques	Rationale	Effect on risk of selection bias
Use blinded recruiters	If recruiters are blind to previous trial allocations, they will be unable to predict the next allocation	Risk of selection bias will be eliminated provided the blinding is maintained
Use simple (unrestricted) randomisation	Recruiters cannot guess the next allocation with any degree of accuracy	Risk of selection bias will be eliminated
If restricted randomisation is used, do not stratify by site of recruitment	The probability of the allocation will depend on previous allocations at other sites, which recruiters are unlikely to have access to, making an accurate guess more difficult	Risk of selection bias will be reduced, but not necessarily eliminated
When randomisation is stratified by site, avoid permuted blocks	Permuted blocks stratified by site will maximise the probability of correctly guessing the next allocation. Using alternative randomisation methods will reduce the probability of correctly guessing the next allocation	Risk of selection bias will be reduced, but not necessarily eliminated
When restricted randomisation is used, stratify by prognostic covariates as well	There is typically less variation in prognoses for patients with the same covariate pattern, making it more difficult for investigators to identify patients with a specific prognosis to enrol into the trial when their preferred treatment is more likely	Risk of selection bias will be reduced, but not necessarily eliminated

*This table assumes that allocation concealment has been appropriately implemented

### Selection bias

For trials that use adequate allocation concealment, selection bias can still be a problem if recruiters can guess the next allocation with greater than 50 % probability. This can occur when restricted randomisation is used. Restricted randomisation involves forcing the treatment groups to be similar in some way: for example, by requiring that a similar number of patients are assigned to each treatment group. The most common methods of restricted randomisation are permuted blocks and minimisation (described below) [16].

Restricted randomisation can increase the risk of selection bias as follows; consider a trial in which patients are randomised using permuted blocks of size 4. This design forces the number of patients in each treatment group to be equal at the end of each block. This means that the imbalance between groups can never be more than two patients. When the imbalance between groups is 1, the recruiter will be able to correctly guess the next allocation with 83 % probability; when the imbalance is 2, this probability increases to 100 %.

### Methods to reduce the risk of selection bias

#### Simple randomisation

Simple randomisation (sometimes also referred to as ‘complete’ or ‘unrestricted’ randomisation) is both the simplest and most effective method to prevent selection bias. Simple randomisation works by assigning each patient to one of the treatment groups with a certain probability (usually 50 %); this probability is the same for every patient, regardless of previous allocations. For example, consider a trial where 15 of the first 20 patients were assigned to the intervention, and only 5 to the control; when the 21st patient presented for randomisation, they would still have an equal chance of being assigned to either treatment group, regardless of the imbalance in numbers. Because the probability is always the same, recruiters will not be able to guess with any accuracy which treatment the patient will be assigned to (as they would essentially be trying to guess the results of a coin-flip); therefore, selection bias cannot occur in this scenario.

In practice, simple randomisation is infrequently used [16, 17], possibly because investigators prefer randomisation methods which provide balance in the number of patients assigned to each treatment group. However, provided the overall sample size is not too small, this lack of balance has only a very small impact on power, and should not be used as a reason to avoid simple randomisation [6]. Therefore, we agree with others that simple randomisation should be used more frequently in practice [8, 17, 18].

#### Do not stratify by site of recruitment if restricted randomisation is used

Despite the appeal of simple randomisation, some form of restricted randomisation is usually employed in practice. In trials with multiple sites of recruitment, the number of patients allocated to each arm can be forced to be similar in two different ways; either by forcing the number within each site to be the same (stratified by site), or by forcing the overall numbers to be the same, regardless of the numbers within each site (not stratified by site).

The risk of selection bias is most pronounced when randomisation is stratified by site of recruitment; that is, when the randomisation procedure is restricted to ensure an equal number of patients are allocated to each treatment group within each site. In this case, the probability of the next allocation depends solely on the previous allocations at that site, which the recruiters may have access to. However, if randomisation is not stratified by site, then the probability of the next allocation will also depend on the previous allocations at all the other sites, which the recruiter is unlikely to have access to.

Therefore, even if restricted randomisation is used, the risk of selection bias can be reduced by not stratifying by site of recruitment. Investigators often stratify by site of recruitment as they are concerned that if between-centre differences are large, a chance imbalance in the number of patients allocated to each treatment within a site could affect results. However, chance imbalances within sites can be accounted for during the analysis by adjusting for site-effects [19–22], and so stratification by site during randomisation is generally not necessary.

#### If randomisation is stratified by site, avoid permuted blocks

Despite the increased risk of selection bias that accompanies stratification by site, it is still commonly used. In some cases, this may be due to administrative or practical reasons: for example when separate randomisation lists must be kept at each site, or when there is only one site and so any restriction on the number of patients assigned to each treatment group is equivalent to stratifying by site. However, it should be noted that in both of these cases, unrestricted randomisation could still be used (e.g. by generating the randomisation list for each site using simple randomisation).

If randomisation is stratified by site, the best method for reducing the risk of selection bias is to avoid the use of permuted blocks, as this has been shown to substantially increase the probability of correctly guessing the next allocation compared to other methods. Common suggestions for improving the performance of permuted blocks are to randomly vary the block size, and to use large block sizes. Although both approaches will help, neither will reduce the risk to an acceptable level [4, 23]. Therefore, permuted blocks stratified by site of recruitment should be avoided.

A preferable alternative is minimisation, which involves allocating the patient to whichever treatment arm minimises the imbalance in a set of baseline covariates. Minimisation can maintain some balance within centres, while also reducing the probability of correctly guessing the next allocation [12]. This is partly because the next allocation at each centre will depend on covariate information for patients from other centres, which recruiters are unlikely to know. Minimisation should only be used with a random element (that is, by assigning patients to the treatment group which minimises the imbalance with a degree of probability, rather than in a deterministic way) [24]. Additionally, centre should be included as a minimisation factor like any other covariate (as opposed to using a stratified approach, where the minimisation procedure is performed separately within each site). Other alternatives to reduce the risk of selection bias when stratifying by site are also available, such as a stratified urn design [25]. However, although these techniques will reduce selection bias compared to permuted blocks, it is still preferable to avoid stratifying by site altogether.

#### If restricted randomisation is used, balance on prognostic covariates as well

When restricted randomisation is used, balancing or stratifying on prognostic factors could help to combat selection bias. This works as follows: consider a trial using permuted blocks. If the recruiter knows that more patients have been allocated to the control, they can guess that the next allocation is likely to be to the intervention. Depending on their beliefs (conscious or subconscious), they may wish to enrol a relatively sick or relatively healthy patient for this allocation. The recruiter therefore needs to be able to find a patient who is relatively sick or healthy, compared to those who have been previously enrolled.

Imagine if the patient’s age and their disease stage have been included as stratification factors during randomisation. Even if the recruiter can guess the next allocation, they still need to be able to find a patient to enrol who is much sicker or healthier than other patients *of the same age and disease stage*. This is clearly a much more difficult task, as there will typically be less variation in prognosis for patients with the same covariate patterns, making it more difficult to identify patients with specific prognoses. This becomes increasingly more difficult the more prognostic covariates that are added to the randomisation procedure (although it should be noted that increasing the number of stratification factors has implications for the analysis as well [16, 26, 27]).

Therefore, when restricted randomisation is used, we would recommend also including prognostic covariates in the randomisation process. However, we caution that this is approach is not a valid reason to use a randomisation method that increases the risk of selection bias, such as stratification by site of recruitment; it is still preferable to use a method of randomisation that completely eliminates the risk of selection bias, such as simple randomisation.

#### Ensure that those enrolling patients are blinded to previous treatment allocations

Even if a trial is unblinded, it may still be possible to blind recruiters (the people who recruit and enrol patients into the trial) [28]. This could be done by using personnel who are not otherwise involved in patient care as recruiters. If they are blinded, they will not be aware of the previous trial allocations and, therefore, will not be able to predict the next allocation.

### Review of published trials

We performed a review of published trials to investigate whether investigators are taking adequate steps to reduce the risk of selection bias. We included parallel group, individually randomized, controlled trials which were not fully blinded. We defined a trial to be not fully blind when at least some trial personnel were not blinded to treatment allocation. This included (but was not limited to) participants, those administering the intervention, those providing medical care apart from the intervention, and those assessing outcomes. We excluded trials which were described as double-blind, stated that everyone involved in the study was blinded, or which used a placebo or sham treatment that was described as being identical to the intervention in terms of appearance. We also excluded pilot and phase I or II trials, as well as articles that reported only secondary analyses.

We included trials that were published in one of four major medical journals in 2010 (*BMJ*, *Journal of the American Medical Association*, *The Lancet*, and *New England Journal of Medicine*). Trials were identified from the electronic table of contents for each journal. One reviewer determined whether trials met the eligibility criteria for all trials identified; a second reviewer assessed this for a subset of trials, and agreement was found to be 100 %.

We extracted data onto a pre-piloted, standardised form. This included information on the blinding status of the trial, whether the randomisation procedure was stratified by site of recruitment (i.e. whether it would be possible for recruiters to keep track of each previous allocation at their site), whether the recruiters were blinded to previous allocations, and details on the randomisation method used. All trials were extracted by two independent reviewers, and disagreements were resolved by discussion.

## Results

We identified 152 eligible trials. General trial characteristics are shown in Table 2. Only 3 (2 %) stated whether those enrolling or recruiting patients were aware of previous allocations (2 were blinded, 1 was unblinded); 149 (98 %) gave no information on whether recruiters were blinded to previous treatment allocations. In most cases, the trial report did not state who was involved in recruiting patients, and whether this person had any other role in the trial, such as delivering the intervention, providing other aspects of patient care, or assessing outcomes.

**Table 2:** The use of methods to reduce the risk of selection bias

	Trials (*n* = 152)
Recruiters blinded? – number (%)	
No	1 (1)
Yes	2 (1)
Not stated	149 (98)
Used simple or restricted randomisation? – number (%)	
Simple randomisation	4 (3)
Restricted randomisation	95 (63)
Not stated	53 (35)
Type of restricted randomisation used – number (%)	
Permuted block	72/95 (76)
Minimisation	21/95 (22)
Other	2/95 (2)
Balanced for prognostic factors after restricted randomisation? – number (%)	
No	42/95 (44)
Yes	53/95 (56)
Randomisation stratified by site of recruitment? – number (%)	
No	41 (27)
Yes	67 (44)
Not stated	44 (29)

**Table 3:** The use of methods to reduce the risk of selection bias in trials that stratified by site of recruitment

	Trials (*n* = 67)
Recruiters blinded? – number (%)	
No	1 (1)
Yes	1 (1)
Not stated	65 (97)
Randomisation method – number (%)	
Stratified permuted block	39 (58)
Minimisation	13 (19)
Other	3 (4)
Not stated	12 (18)
Block sizes stated? – number (%)	
No	16/39 (41)
Yes	23/39 (59)
Were block sizes random? - number (%)	
No	16/39 (41)
Yes	6/39 (15)
Not stated	17/39 (44)
Largest block size used – median (IQR)	8 (4 to 11)
Was minimisation deterministic? – number (%)	
No	1/13 (8)
Yes	0/13 (0)
Not stated	12/13 (92)
Balanced for prognostic factors? – number (%)	
No	27 (40)
Yes	40 (60)
Number of prognostic factors balanced – median (IQR)	2 (1 to 3)

*IQR*, interquartile range

Only 4 trials (3 %) used simple randomisation; the rest used either restricted randomisation (*n* = 95, 63 %), or did not state the method of randomisation (*n* = 53, 35 %). Of the trials using some form of restricted randomisation, most used permuted blocks (n = 72, 76 %) or minimisation (*n* = 21, 22 %). Overall, 67 (44 %) of trials stratified randomisation by site of recruitment; 41 (27 %) did not, and 44 (29 %) did not provide this information.

### Results for trials which stratified by site of recruitment

Amongst the 67 trials which stratified by site of recruitment, 65 (97 %) did not provide information on whether recruiters were blinded (Table 3). Most trials (*n* = 39, 58 %) used stratified permuted blocks; 13 (19 %) used minimisation, 3 (4 %) used another method of randomisation, and 12 (18 %) did not state which method they used.

Only 23 of the 39 trials (59 %) which used permuted blocks stated the block size(s), and only 6/39 (15 %) reported using random block sizes. Seventeen out of thirty-nine 17/39 (44 %) did not state whether block sizes were random or not. Most trials used small block sizes (median 8, interquartile range (IQR) 4 to 11). Of the 13 trials using minimisation, 12 (92 %) did not state whether it was deterministic or not.

## Discussion

Selection bias can subvert the randomisation process in RCTs, leading to biased estimates of treatment effect and misleading conclusions. For this reason, allocation concealment is regarded as an essential component of RCTs. However, selection bias can still occur even with adequate allocation concealment if the method of randomisation is poorly chosen.

Our review found that very few trials used techniques that would eliminate the risk of selection bias, such as simple randomisation (3 %) or blinding of recruiters (1 %). Most trials used some form of restricted randomisation (63 %), and many trials were stratified by site of recruitment (44 %). Furthermore, a substantial proportion of trials did not provide adequate information on whether randomisation was restricted (35 %) or whether it was stratified by site (29 %), effectively preventing readers from assessing the risk of selection bias. A comparison to Hewitt and Torgerson’s review of trials published in 2002 [17] indicates that reporting on the method of randomisation has not improved in the 8 years between reviews (method of randomisation unclear 34 % in 2002 versus 35 % in 2010), while the use of simple randomisation may have decreased (9 % in 2002 versus 3 % in 2010).

These findings indicate that a substantial proportion of unblinded trials are at risk of selection bias. This is surprising, given the relative simplicity with which methods to reduce this risk can be implemented. For example, simple randomisation is by far the easiest method of randomisation to implement, and has little effect on trial organisation or the statistical analysis. Likewise, adjusting for site of recruitment in the analysis rather than stratifying on it during randomisation is also relatively straightforward, and typically has no adverse impact on results. Similarly, permuted blocks can often be replaced by allocation methods which do not substantially increase the probability of correctly guessing future allocations, such as minimisation with a random element or urn randomisation.

We therefore suggest that investigators conducting unblinded trials (or double-blinded trials where the blinding may not be entirely effective) should choose randomisation methods that reduce the risk of selection bias. This primarily involves simple randomisation, or avoiding the use of stratification by site of recruitment if restricted randomisation is employed. As discussed in the Standard Protocol Items: Recommendations for Interventional Trials (SPIRIT) guidelines, details of restricted randomisation schemes should not be shared with trial investigators, as this information could make it easier to correctly guess the next treatment allocation [29]. However, it is important that the details of the randomisation procedure is fully reported in the trial publication, as this will enable readers to judge the risk of bias. This description should include whether stratification was employed, which stratification factors were used, the block sizes (and whether they were random) for permuted blocks, and whether a random component was used (and its size) for minimisation. It would also be helpful for investigators to report on who was involved in recruiting and enrolling patients into the trial, whether they were blinded to treatment allocation, and whether they had any other role in the trial, such as delivering the intervention, providing other aspects of medical care, or assessing outcomes.

## Conclusion

The risk of selection bias could not be ascertained for most trials due to poor reporting. Many trials which did provide details on the randomisation procedure were at risk of selection bias due to a poorly chosen randomisation methods. Investigators should choose randomisation methods which eliminate or reduce the risk of selection bias.

## Abbreviations

IQR: interquartile range
RCT: randomised controlled trial
SPIRIT: Standard Protocol Items: Recommendations for Interventional Trials
